# Prevalence of anal high-risk human papillomavirus infection, cytologic and histologic abnormalities among Rwandan women living with human immunodeficiency virus

**DOI:** 10.4102/ajlm.v15i1.3116

**Published:** 2026-10-13

**Authors:** Gad Murenzi, Hae-Young Kim, Jean Paul Mivumbi, Josephine Gasana, Faustin Kanyabwisha, Thierry Z. Muvunyi, Samer Khader, Gallican Kubwimana, Qiuhu Shi, Marcel Yotebieng, Leon Mutesa, Philip E. Castle, Kathryn M. Anastos, Joel M. Palefsky

**Affiliations:** 1 Department of Pathology, College of Medicine and Health Sciences, University of Rwanda, Kigali, Rwanda; 2 Department of Research, Research for Development, Kigali, Rwanda; 3 Department of Research, Goodlife Access, Kigali, Rwanda; 4 Department of Public Health, New York Medical College, Valhalla, New York, United States; 5 Biostatistics and Research Design Center, Boston Children’s Hospital, Boston, Massachusetts, United States; 6 Department of Pathology, King Faisal Hospital, Kigali, Rwanda; 7 Department of Pathology, University of Pittsburgh Medical Center, Pittsburgh, Pennsylvania, United States; 8 Department of General Internal Medicine, Albert Einstein College of Medicine, New York, New York, United States; 9 Center for Human Genetics, College of Medicine and Health Sciences, University of Rwanda, Kigali, Rwanda; 10 Division of Cancer Prevention, National Cancer Institute, National Institutes of Health, Bethesda, Maryland, United States; 11 Department of General Internal Medicine, Faculty of Medicine, Albert Einstein College of Medicine, New York, New York, United States; 12 Department of Medicine, University of California San Francisco, San Francisco, California, United States

**Keywords:** anal high-risk human papillomavirus, anal cytology, anal precancer, women living with HIV, Rwanda

## Abstract

This cross-sectional pilot study assessed 51 Rwandan women living with HIV (WLWH) with cervical high-risk human papillomavirus (hrHPV; *n* = 25) but < CIN2 or high-grade squamous intraepithelial lesions (HSIL) (CIN2+), due to higher anal hrHPV risk. Anal swabs underwent hrHPV genotyping and cytology. High-resolution anoscopy-guided biopsy was performed on clinically visible lesions. Mean age was 43.8 years. Anal hrHPV prevalence was 35.3% (95% confidence interval: 22.4–49.9). Cytology was negative (34/51 = 66.7%), unsatisfactory (10/51 = 19.6%), atypical squamous cells of undetermined significance (4/51 = 7.8%), low-grade squamous intraepithelial lesions (2/51 = 3.9%), and atypical squamous cells, cannot exclude HSIL (1/51 = 2.0%). Histology showed low-grade SIL in 21.6% and HSIL in 2.0%. Anal cytologic abnormalities were associated with hrHPV (*p* = 0.032); no cervical–anal concordance was observed. Findings support further research into anal cancer prevention among high-risk WLWH.

**What this study adds:**

Given the paucity of data on anal hrHPV in Rwanda, this study provides the first data about anal hrHPV, cytologic abnormalities and HSIL among WLWH. These preliminary findings will inform further research and may support future exploration of integrating anal cancer screening research within well-established cervical cancer screening platforms, pending larger confirmatory studies.

## Introduction

Women living with HIV (WLWH) face a significantly higher burden of high-risk human papillomavirus (hrHPV) infection than HIV-negative women.^1^ High-risk human papillomavirus types are the causative agents of cervical and anal cancers, and their prevalence remains high despite the scale-up of antiretroviral therapy. While antiretroviral therapy improves immune function and reduces the incidence of many AIDS-related malignancies, hrHPV-related cancers such as anal cancer have not seen the same decline.^2^ In fact, WLWH are up to 15 times more likely to develop anal cancer compared to HIV-negative women.^3^

Evidence from the United States, including the Anal Cancer HSIL Outcomes Research (ANCHOR) study, has shown that treatment of anal high-grade squamous intraepithelial lesions (HSIL) significantly reduces the risk of anal cancer in people living with HIV.^4^ Based on such findings, the Centers for Disease Control and Prevention issued the first formal recommendations for anal cancer screening in people living with HIV.^5^ Recommended tools include anal hrHPV DNA testing and cytology, with high-resolution anoscopy (HRA) and biopsy of suspicious lesions for diagnosis and treatment. For WLWH in sub-Saharan Africa (SSA), platforms developed for cervical cancer screening may offer opportunities for integrated screening, though data to support such efforts are currently limited. Furthermore, a systematic review by Lin et al. found anal HPV16 in 46% of WLWH with HPV16 cervical infections, suggesting a strong correlation between cervical and anal hrHPV infections. They also reported a prevalence of anal HSIL of 25% in cervical HPV16-positive women.^6^

Given the differences in sexual behaviour among populations in SSA, it is important to compare anal hrHPV and HSIL with cervical hrHPV and HSIL as self-transmission is a potential mode for anogenital human papillomavirus (HPV) infection. That comparison will provide important information on whether cervical hrHPV and HSIL are predictors of anal hrHPV and HSIL in SSA with potential for integrated preventive strategies. Therefore, we aimed to determine the prevalence of anal hrHPV genotypes and squamous intraepithelial lesions (SIL) in Rwandan WLWH who have a history of cervical hrHPV infection or cervical HSIL.

## Methods

### Ethical considerations

The protocol was reviewed and approved by the Rwanda National Ethics committee (RNEC), initially approved on 24 January 2019 with reference number 0019/RNEC/2019 and then reviewed and approved annually with the current approval on 21 March 2025 with reference number RNEC740/2025.

### Study design, population and setting

This cross-sectional pilot study was conducted between 30 January 2019 and 29 March 2019 at Rwanda Military Teaching Hospital in Kigali, Rwanda, as part of a larger cervical cancer screening project. Eligible participants were WLWH aged 18 years or older who had undergone cervical cancer screening and had either: (1) confirmed cervical HSIL or cervical intraepithelial neoplasia grade 2 or worse (≥ CIN2+) on biopsy, or (2) cervical hrHPV-positive results with pathology < CIN2.

### Data collection methods

With the participant in the left lateral decubitus position, a water-moistened Dacron swab was inserted into the anal canal until the rectal vault was hit and then rolled clockwise and counter-clockwise as it was withdrawn for at least 30 s. The swab was then vigorously shaken in PreservCyt. A digital anorectal examination was then performed and a plastic anoscope inserted into the anal canal to apply 5% acetic acid for 2 min and then HRA performed by examining the entire anal canal with continuous application of 5% acetic acid and strong Lugol’s iodine was also applied. Biopsies were taken from any area clinically suspicious for a lesion and placed in 10% formalin; participants with a normal HRA impression did not undergo biopsy. No participant had undergone prior anal cancer screening. HIV-related clinical data (CD4+ cell count and viral load) were abstracted from participants’ medical records.

### Laboratory testing

#### Anal HPV testing, cytology and pathology

Human papillomavirus testing was performed using the AmpFire HPV genotyping assay (Atila Biosystems Inc., Mountain View, California, United States), an isothermal nucleic acid amplification-based, real-time fluorescence detection of 15 HPV genotypes (16, 18, 31, 33, 35, 39, 45, 51, 52, 53, 56, 58, 59, 66, and 68) individually in four reaction tubes as previously described.^7^

Monolayer liquid cytology slides were prepared from the PreserveCyt bottles using the Hologic Thinprep 2000 cytology processor following the laboratory’s standard operating procedures. Slides were then read by certified cytotechnologists and pathologists following standard practice. Biopsies were processed and stained using hematoxylin and eosin and then slides read by at least two pathologists to confirm the diagnosis and a consensus reached in case the two pathologists disagreed.

### Data analysis

Descriptive statistics summarised participant characteristics and laboratory results. Wilcoxon rank sum and Fisher’s exact tests were used to compare groups. Bowker’s test assessed agreement between anal and cervical hrHPV results. A *p*-value < 0.05 was considered statistically significant.

## Results

The mean age of participants was 43.8 (standard deviation [s.d.] = 7.2) years. Anal hrHPV prevalence was 35.3% (95% confidence interval: 22.4–49.9). Most participants had negative anal cytology (34/51, 66.7%); 10/51 (19.6%) had unsatisfactory results. Cytologic abnormalities included atypical squamous cells of undetermined significance in 4/51 (7.8%), low-grade squamous intraepithelial lesion in 2/51 (3.9%), and atypical squamous cells, cannot exclude HSIL in 1/51 (2.0%). Of the 51 participants, 35 (68.6%) underwent biopsy based on suspicious HRA findings. On histology, 9/35 (25.7%) had low-grade squamous intraepithelial lesion and 1/35 (2.9%) had HSIL; 25/35 (71.4%) had negative biopsy results. The remaining 16 participants (31.4%) had a normal HRA impression and did not undergo biopsy.

Comparisons of sociodemographic characteristics, HIV, clinical and laboratory variables by anal hrHPV status are shown in Table 1. Those with abnormal anal cytology (*p* = 0.032) were more likely to have anal hrHPV. Pairwise comparisons of cervical screening, cervical colposcopy, and anal hrHPV test results classified in hierarchical categories of hrHPV according to cancer risk (HPV16 positive, else HPV16 negative but HPV18 positive, else HPV16 and HPV18 negative but positive for other hrHPV types, else hrHPV negative) are shown in Table 2. There was concordance for the cervical screening and colposcopy visit hrHPV but no concordance for anal hrHPV and either the cervical screening and colposcopy visit hrHPV. There were no significant differences for sociodemographic, clinical and laboratory characteristics by CIN2+ presence versus not as shown in Online Supplementary Table 1.

**TABLE 1:** Socio-demographic, clinical and laboratory characteristics by anal hrHPV status at Rwanda Military Teaching Hospital, Kigali, Rwanda, 30 January 2019 to 29 March 2019 (*N* = 51).

Variable	hrHPV-negative (*N* = 33)		hrHPV-positive (*N* = 18)		*p*-value
	*n*	%	*n*	%	
Median age (years)	41.0	-	38.500	-	0.2500
Median income (Rwandan Francs)	30 000.0	-	37 500.0	-	0.0190
Median age at sexual debut (years)	18.0	-	18.0	-	0.2700
Median age at first child (years)	20.0	-	20.0	-	0.2700
Cervical hrHPV-negative	5.0	55.6	4.0	44.4	-
Cervical hrHPV-positive	28.0	66.7	14.0	33.3	0.7029
Cervical biopsy negative or LSIL	10.0	62.5	6.0	37.5	0.8974
Cervical biopsy HSIL	10.0	52.6	9.0	47.4	-
HRA impression normal	15.0	75.0	5.0	25.0	-
HRA impression LSIL	1.0	50.0	1.0	50.0	-
HRA impression HSIL	7.0	46.7	8.0	53.3	0.2045
Anal cytology unsatisfactory	7.0	70.0	3.0	30.0	-
Anal cytology negative	25.0	73.5	9.0	26.5	-

HRA, high-resolution anoscopy; hrHPV, high-risk human papillomavirus; LSIL, low-grade squamous intraepithelial lesions; HSIL, high-grade squamous intraepithelial lesions; hrHPV, high-risk human papillomavirus

**TABLE 2:** Pairwise comparisons of cervical screening, cervical colposcopy, and anal specimen hrHPV testing results classified in hierarchical categories of hrHPV according to cancer risk (HPV16 positive, else HPV16 negative but HPV18 positive, else HPV16 and HPV18 negative but positive for other hrHPV types, else hrHPV negative) at Rwanda Military Teaching Hospital, Kigali, Rwanda, 30 January 2019 to 29 March 2019.

Category	HPV16 (cell%)		HPV18 (cell%)		Other hrHPV (cell%)		hrHPV negative (cell%)		*p*-value*
	*n*	%	*n*	%	*n*	%	*n*	%	
**Cervical Screening hrHPV Category: Colposcopy cervical hrHPV category**									< 0.0001
HPV16	**26**	**51.0**	0	0.0	0	0.0	2	3.9	-
HPV18	1	2.0	**1**	**2.0**	1	2.0	0	0.0	-
Other hrHPV	0	0.0	0	0.0	**11**	**21.6**	2	3.9	-
hrHPV negative	1	2.0	0	0.0	1	2.0	**5**	**9.8**	-
**Cervical Screening hrHPV Category: Anal hrHPV category**									0.8477
HPV16	**5**	**9.8**	0	0.0	1	2.0	2	3.9	-
HPV18	1	2.0	**0**	**0.0**	1	2.0	0	0.0	-
Other hrHPV	3	5.9	0	0.0	**3**	**5.9**	2	3.9	-
hrHPV negative	19	37.3	1	2.0	8	15.7	**5**	**9.8**	-
**Colposcopy Cervical hrHPV Category: Anal hrHPV category**									0.3120
HPV16	**5**	**9.8**	1	2.0	0	0.0	2	3.9	-
HPV18	1	2.0	**0**	**0.0**	1	2.0	0	0.0	-
Other hrHPV	3	5.9	1	2.0	**4**	**7.8**	0	0.0	-
hrHPV negative	19	37.3	1	2.0	8	15.7	**5**	**9.8**	-

Note: The bolded text highlights concordance for those categories for the pairwise comparison.

HPV, human papillomavirus; hrHPV, high-risk human papillomavirus.

* , *p*-value from the Bowker’s test.

## Discussion

This study aimed to determine the prevalence of anal hrHPV, cytologic and histologic abnormalities among Rwandan WLWH with cervical precancer or hrHPV. Despite being a small pilot study, it is among the first studies to explore those outcomes in Rwanda and in SSA in a population among the highest at risk of anal hrHPV and related disease. We found a relatively high prevalence (35.3%) of anal hrHPV albeit lower proportions of cytologic abnormalities and biopsy-confirmed SIL. These findings are different from those found in a study done in South Africa which found higher proportions of anal hrHPV and cytologic and histologic abnormalities.^8^

Although our study included high-risk women (with cervical hrHPV or biopsy-confirmed CIN2+), the prevalence of anal hrHPV among these Rwandan WLWH was higher than that found among Rwandan men who have sex with men (MSM).^9^ However, the prevalence in our study and the South African studies is lower than a pooled analysis conducted by Lin et al. which found a prevalence of 62% among WLWH.^6^ Various factors have been found to be associated with anal hrHPV including younger age, number of lifetime sex partners, genital warts and lower CD4+ cell counts in the South African study,^8^ although lower CD4+ cell counts were non-significant. Nonetheless, we did not find any differences by age in our study and we found a similar non-significant association to the South African study, between lower CD4+ cell counts and anal hrHPV positivity, possibly due to the small sample size.

Given the similarity between HPV-related anal and cervical disease and their prevention strategies, existing cervical cancer screening infrastructure may eventually offer a platform to support anal cancer screening, as demonstrated by the ANCHOR study^4^ and Centers for Disease Control and Prevention recommendations.^5^ Additionally, the close anatomic proximity between the cervix and anus implies a potential risk for self-inoculation from one site to the other, further motivating comparison of hrHPV infection at both sites. Our comparison of anal and cervical hrHPV showed no statistically significant concordance, except for types other than HPV16/18 showing a tendency to co-occur at both anatomic sites. This is somewhat different from other studies which showed significant agreement between hrHPV infection at both anatomic sites.^6,8^

### Limitations and strengths

Our study has several important limitations. First, this was a small pilot study of only 51 women and was not powered to detect statistically significant associations; all findings should therefore be interpreted as exploratory and hypothesis-generating. Second, the enrolment of WLWH with at least one known cervical outcome (hrHPV positivity or CIN2+) constitutes a selection bias, and our findings cannot be generalised. Third, 10/51 (19.6%) of participants had unsatisfactory anal cytology results, likely due to insufficient cellularity of swab specimens, which may have led to underestimation of cytologic abnormality prevalence. Despite these limitations, our study adds to the currently limited body of evidence in Rwanda and SSA about anal hrHPV infection, cytologic and histologic abnormalities and their relationship with cervical hrHPV and disease among WLWH, providing a foundation for future studies.

### Conclusion

Our pilot study found a high prevalence of anal hrHPV among Rwandan WLWH with cervical HSIL or hrHPV infection, underscoring the need for larger studies to investigate anal cancer risk and prevention in high-risk populations in Rwanda and SSA. These preliminary findings also support exploration of the feasibility of integrating anal cancer screening research within existing cervical cancer prevention platforms.
